# Onset Rivalry: Brief Presentation Isolates an Early Independent Phase of Perceptual Competition

**DOI:** 10.1371/journal.pone.0000343

**Published:** 2007-04-04

**Authors:** Olivia Carter, Patrick Cavanagh

**Affiliations:** 1 Vision Sciences Laboratory, Harvard University, Cambridge, Massachusetts, United States of America; 2 Brain Research Institute, Melbourne, Australia; 3 Laboratoire de Psychologie de la Perception, Université de Paris Descartes, Paris, France; University of Minnesota, United States of America

## Abstract

When the left and right eyes are simultaneously presented with different images, observers typically report exclusive awareness of only one image. This phenomenon is termed binocular rivalry, reflecting the fact that the dominant image alternates every few seconds in a cycle of perceptual competition that continues indefinitely. Despite the apparent continuity in perceptual switching, we now demonstrate that the initial “onset” period is fundamentally different to all subsequent rivalry epochs. Using brief intermittent presentations, rivalry dominance shows strong biases such that the same target is perceived with each successive stimulus onset. These biases remain consistent within any given location, but vary across the visual field in a distribution that is stable over multiple weeks but highly idiosyncratic across observers. If the presentation exceeds ∼1sec at any location, however, the very different and much more balanced alternations of sustained binocular rivalry become apparent. These powerful onset biases are observed with brief intermittent presentations at a single location or with continual smooth motion of the targets. Periods of adaptation to one of the rivaling targets induced local switches in dominance to the non-adapted target. However, these effects were generally limited to the spatial site of adaptation and had less influence over each subsequent cycle of the target. We conclude that onset rivalry is independent of sustained rivalry and cannot be explained by local regions of monocular dominance or memory of past perceptual history, but rather reflects low-level, spatially localized factors that are stable over periods of weeks. These findings suggest that brief presentation paradigms are inappropriate for their current use in studies of the mechanisms underlying sustained rivalry. However, brief presentations are ideal for investigating early stages of perceptual competition.

## Introduction

What happens when the brain is presented with ambiguous visual input? For the most part, the visual system tends to “choose” one valid interpretation at the expense of the alternatives. This winner-takes-all strategy is illustrated by popular illusions such as the Necker cube and binocular rivalry. In the case of binocular rivalry, two different images are presented simultaneously to the corresponding location in each of the two eyes. Under these conditions, the observer will be more likely to see one image rather than a superposition of the two. After a few seconds, the previously suppressed image will become dominant and then continue to cycle between suppression and dominance in a quasi-regular fashion [1], [2].

Due to the dramatic switches in perception between complete dominance and total suppression of an image, binocular rivalry has attracted considerable attention. Most notably it has been used as a tool to investigate the neural correlates of conscious experience [3], perceptual organization [4], feature binding [5] and the limits of unconscious visual processing [6]. Despite decades of research, the neural mechanisms underlying binocular rivalry remain debated. Originally binocular rivalry was believed to result directly from mutual inhibition between competing monocular neurons-the suppression and dominance phases being viewed as two sides of the same coin. Since then, new evidence has led to the proposal of hierarchical models that depend on multiple distributed processes. These models suggest that the three main components of rivalry (i.e. the generation of transitions between dominant percept as well as the maintenance of perceptual dominance and suppression) may all involve quite distinct mechanisms[2], [7]. Despite the complexity of these models and the increasing sophistication of psychophysical experiments, little attention has been paid to differences between successive periods of rivalry dominance. After early work by Wolf (1983), showing that there was an initiation period of approximately 150ms-during which time perceptual fusion was experienced-it has generally been accepted that the initial dominance period of rivalry is fundamentally identical to those driving every subsequent dominance state. Recently, however, unique properties of the initial dominance period have come to light. Specifically, results by Chong and Blake (2006), show that attention may have a greater influence on the initial selection of dominance compared to the subsequent maintenance of the suppression/dominance phases. Here we would like to extend this claim of independence further–beyond the influence of attention. We would like to claim that the initial dominance state is fundamentally different to the subsequent periods of dominance and suppression that occur during sustained rivalry. This period that we have termed “onset rivalry” refers to the time between stimulus onset and the first transition.

## Results

### Experiment 1& 2

During the period of “normal” sustained rivalry the frequency of rivalry transitions can be manipulated through changes to stimulus parameters such as contrast, luminance, motion and salience [8], [9]. Alternatively the stimulus can be moved continually around the observer's visual field [10] or intermittently removed [11]. If the removal period is less than 200–300msec, the two images will continue to switch dominance at the original rate [12]. However, a number of recent studies have shown that the perceptual switching will slow down, or even stabilize completely, if the removal duration extends to 2-3sec [11], [13], [14]. Leopold et al., (2002) argued that this effect could not be explained by a “reset” mechanism or the existence of “permanent perceptual biases” because perceptual dominance was more likely to follow the dominance experienced at the end of the previous presentation, even if there had been a perceptual switch during that presentation.

**Figure 1:**
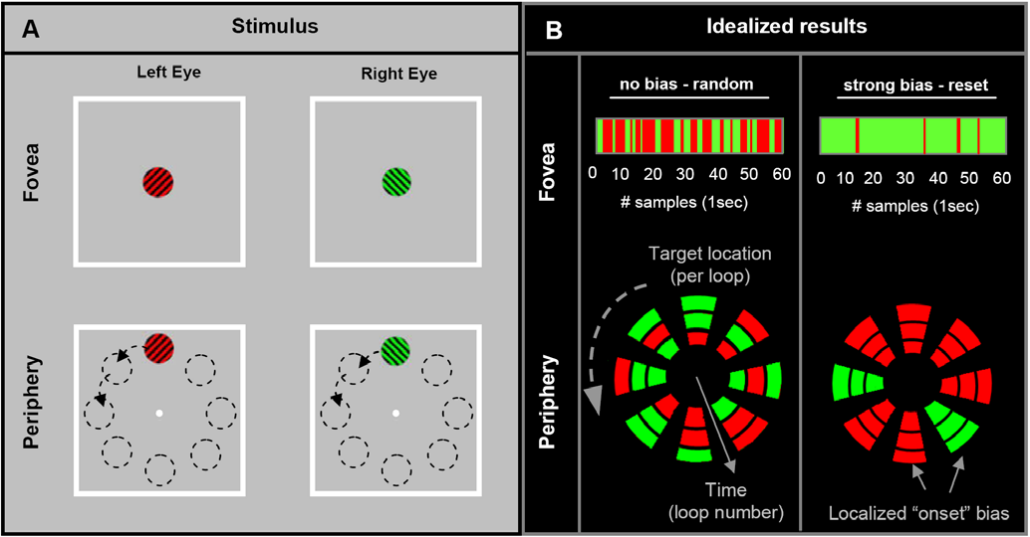
Illustration of stimulus and data presentation method. (A) In experiment 1, binocular rivalry was induced with foveal presentation of red and green orthogonal gratings to the left or right eye respectively. The rivaling targets were red and green sinusoidal grating patches that subtended 2.5° of visual angle. The gratings had a spatial frequency of 1.6 cycles/deg oriented obliquely at either 45° or 135°. Targets were presented for either 60sec of continual presentation or for 60×1sec presentations over 600 sec (i.e. 1sec on 9sec off). In experiment 2, the same targets were presented in one of 8 locations spaced equally around a circular path with a radius of 4.5° around a 0.3° central fixation point. During the intermittent stimulus conditions targets were presented for 1sec with an inter-stimulus interval 250ms (all 8 locations visited every 10sec). In both conditions the targets were presented on a gray background within a 13.5° white square frame 0.2° thick. (B) Schematic illustration of results for an idealized case of random and strongly biased perceptual dominance (note that any switch in perception is only temporary, suggesting the dominance reflects an onset bias rather than stabilization). For foveal rivalry the data are graphically represented as a sequence of 60 adjacent colored bars corresponding (from left to right) with the perceptual dominance for each of the 60×1sec presentation (in the intermittent case the gap interval is not depicted). In experiment 2, perceptual dominance at each location is illustrated by a color patch corresponding to each of the 8 peripheral target locations. Each successive target loop is represented in increasingly outward rings. In this way, each color patch represents the subject's perceptual dominance at a single point in time and space for the entire trial. The left column represents idealized case for random allocation of dominance, while the right column shows complete localized biases.

We were interested to investigate whether permanent perceptual biases could ever be observed at rivalry onset. It was reasoned that demonstration of an “onset bias” would require: 1) evidence of a dominant perceptual state-the near exclusive dominance of the same target across each successive stimulus presentation, 2) evidence against perceptual stabilization-any occurrence of a switch in perceptual dominance would have to be temporary, reverting quickly to the originally dominant state (figure 1B upper row). To test this possibility, our initial experiment used brief (1sec) presentation times and long (9sec) interstimulus intervals (ISIs). Perceptual dominance during this intermittent presentation was then compared against continuous rivalry presentation. In both conditions the orthogonally oriented red and green grating targets were presented in the fovea for a total of 60sec (figure 1A upper row).

**Figure 2:**
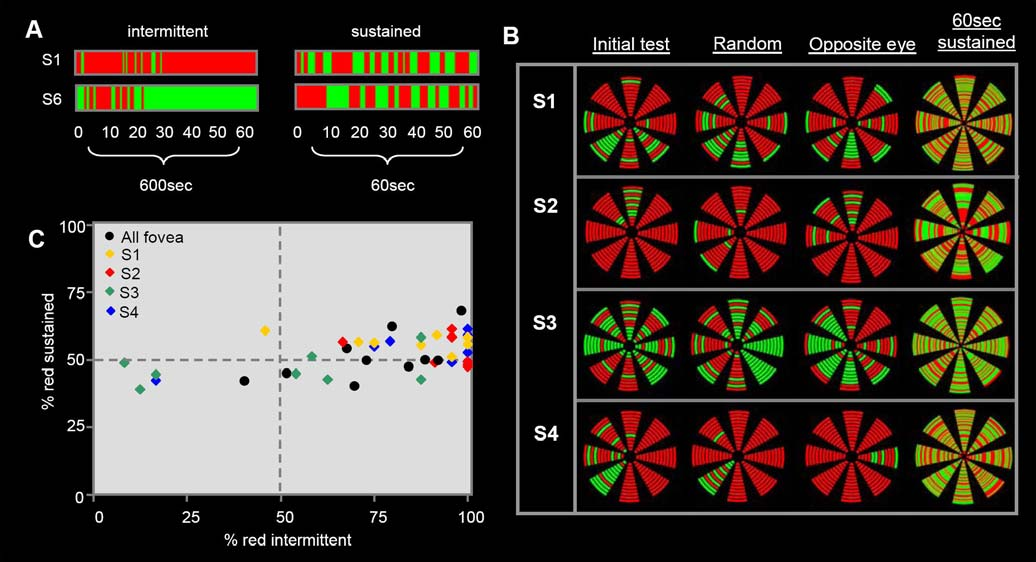
Results from experiment 1 and 2. (A) The pattern of perceptual dominance for two representative subjects during foveal presentation of rivalry targets. The left column depicts results for 60 intermittent 1sec presentations (9 sec inter-stimulus intervals). During this condition there was no evidence of perceptual stabilization. Subjects reported large perceptual biases that were broken by brief intermittent dominance of the alternative targets. In contrast, no biases in perceptual dominance were observed during 60sec of sustained target presentation. (B) Data from all 4 subjects tested with the peripheral presentation in experiment 2a–d. The left column shows results from the initial test of sequential (clockwise or counterclockwise) presentation. During the 2min trial duration the targets cycled through the same 8 locations 12 times (each wedge represents a single location in space, with time represented in radial distance from the center ring). In this condition, perceptual dominance shows strong localized biases. Similar biases were also observed when the order of presentation was randomized (columns 2^nd^ and 3^rd^ from the left respectively), however, the relative locations of the biases were not necessarily conserved. No systematic biases were observed during 60sec of sustained presentation at each of the 8 locations (4^th^ column). (C) A plot of the proportion of red dominance reported during intermittent (x-axis) and sustained target presentation (y-axis) for the foveal (black circles) and 8 peripheral target locations (color diamonds correspond to the 4 subjects tested in experiment 2). Despite very little bias in dominance during sustained viewing subjects reported large biases during intermittent viewing across all target locations.

In support of the existence of an onset bias, results showed that there were large biases in the reported dominance of the two targets during intermittent presentation (60×1sec presentations over a 600sec trial). Furthermore, on the rare occasions that the non-dominant target was perceived, the dominance quickly returned to the target with the strongest perceptual bias. However, when the same targets were presented continuously for 60sec these large biases in dominance were no longer seen (the two targets dominated for approximately equal durations) (Figure 2A and 2C-black circles).

Two recent studies explored the factors influencing the stabilization effect originally described by Leopold et al., (2002). Together they showed that perceptual memory is location specific [14] but not necessarily eye specific [13]. Therefore, we were interested to see if the onset bias observed in the fovea also exists in other locations of the visual field and whether the bias was specific to the eye-of-presentation or a predictable/repeated target path. To explore these questions in experiment 2a, the same targets used in the foveal experiment were now presented in a sequence through eight different locations spaced equally around the periphery of the visual field (Figure 1A lower panel). The rivalry targets were presented once at each location for a period of 1sec. There was an inter-stimulus interval of 0.25sec such that all eight locations were visited once every 10sec with the targets presented sequentially in order around a clockwise or counterclockwise trajectory for 2min (each location was visited twelve times).

In line with the foveal results described in experiment 1, during peripheral presentation, rivalry dominance was again reported to have very strong and consistent biases at each location. Surprisingly, however, the bias varied at different locations. For example, one target might dominate seven of the eight locations, but the dominance would be reversed in the eighth location (Figure 2B column 1). Given the surprising nature of this observation we were interested to understand the factors influencing the local onset bias.

One possibility was that onset rivalry may depend on the sequence of stimuli rather than their locations such that, for example, dominance begins with one target but switches on the eighth stimulus to the alternative target. To test this, subjects were presented the same rivalry targets used in experiment 2a, however, this time the order of presentation was randomized rather than sequential. Illustrated in column 2 of figure 2B, the same location specific onset biases were observed even when there was no common path trajectory.

Another potential explanation is that local biases reflect localized regions of monocular dominance. Given that binocular rivalry involves competition between images presented to the two eyes, it is important to rule out the possibility that the local onset biases result from regions of monocular dominance due to some sort of inherent variability in the retina or low-level cortical areas. To test this possibility, subjects from experiment 2a were retested after the eye of presentation was switched for each target (all other stimulus parameters were identical to those used in experiment 2a). Under these conditions onset rivalry still shows clear location specific biases. The interesting thing to note is that the observed biases are different but not complementary/opposite to those reported under the original conditions (Figure 2B column 3). While this result does not rule out the existence of local monocular factors, it suggests that onset rivalry bias is not simply the result of local zones of monocular dominance. To further investigate the existence of inherent local visual field biases, in experiment 2d subjects were presented with 60sec of continual rivalry in each of the eight locations (Figure 2B column 4). Consistent with the results from the foveal presentation, the large biases found in the initial stage did not carry over to the periods of sustained viewing (Figure 2C colored shapes) where the biases collapsed to a much smaller range showing little relation to the onset biases. For example, across the eight locations for each of the four subjects, the regressions between the onset and sustained biases showed slopes (ß) that were much smaller than 1 (a slope of 1 would be expected if the two reflected the same rivalry) and that were positive for two subjects but negative for the other two (S1: r^2^ = 0.25, ß = −0.8; S2: r^2^ = 0.10, ß = −0.16; S3: r^2^ = 0.15, ß = 0.07; S4 r^2^ = 0.42, ß = 0.14, df = 6). Using a two tailed t-test, none of these correlations was found to be significant. In the foveal condition, the correlation approached but did not reach significance (r^2^ = 0.38, ß = 0.29, p = 0.058, df = 8). Given the small number of subjects and locations tested, it is difficult to determine the exact relation between the onset and sustained biases. However, given the small slopes of the relation between the two (negative in two cases and significantly smaller than a slope of 1 in all cases p<0.001), we conclude that there is a large degree of independence between the dominance properties of rivalry seen at onset and that seen during sustained viewing. More importantly, the lack of any obvious correlation can be taken as further evidence that the “onset” biases observed were not reflecting general inherent biases in monocular dominance.

In summary, results from the first 2 experiments show that dominance at rivalry onset is determined by location specific factors that are different from those biasing dominance during sustained rivalry. The biases cannot be explained by local variability in monocular dominance or the build up of rivalrous inhibitory interactions over the target path history.

### Experiment 3, 4 & 5

Together the results from experiment 1 and 2 suggest the existence of an onset suppression that is quite distinct from the subsequent state of binocular rivalry. Experiment 3 explored whether similar local biases occur during sustained presentation of a continually moving target. The one study that has previously investigated this question suggests that continuous motion of a rivalry target can slow down and even stabilize rivalry alternations [10]. However the authors of that study specifically claim that their results show no evidence of location specificity. This claim stands in contrast to our findings with the discrete intermittent presentation used in experiment 2a–c. In the original study, stabilization was found to increase with the speed of target motion. Accordingly, four speeds were chosen for this study (0.2Hz, 0.1Hz, 0.05Hz, 0.01Hz) such that the target would return to the same location every 5, 10, 20 or 100sec. Apart from these differences in motion speed and the continual presentation of the targets, all other properties of the stimulus were consistent with experiment 2a.

**Figure 3:**
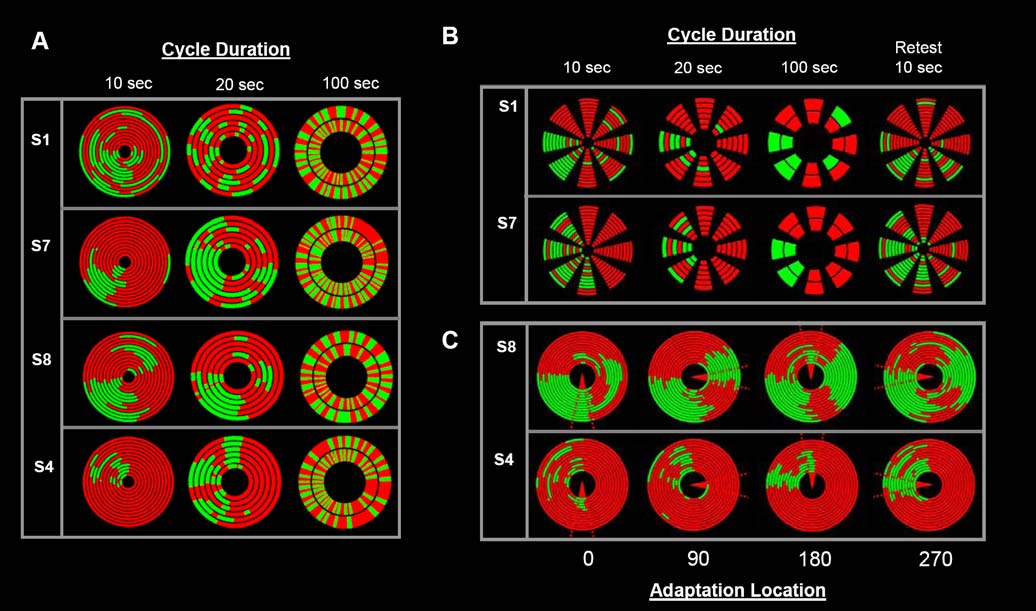
Results from experiment 3, 4 and 5. (A) Data from all 4 subjects tested with 3 different speeds of smooth motion of the target around a peripheral trajectory (the same radial distance used in experiment 2). The left column shows smooth motion at a cycle speed of one loop every 10sec, a rate equivalent to experiment 2a–c. Throughout the 2min trial strong localized biases were reported consistently across all 12 cycle loops. The 2^nd^ and 3^rd^ column show a clear decrease in the strength and location specificity of perceptual bias as the target speed slows down to 20 and 100sec per loop. (B) In experiment 4, the 1sec presentation (identical to experiment 2a–c) was used to determine whether the reduction in bias with slower speeds was related to increases in local presentation duration or the elapsed duration between successive presentations at a single location. Data from 2 (of 4) subjects illustrate that localized biases exist independent of the elapsed duration between successive presentations. Local biases are still observed if the inter-stimulus interval is extended to allow cycle rates of 20 and 100sec and at retest 2 weeks later. (C) 2 subjects were tested with 60sec of adaptation to the dominant target (red in both cases) at 4 locations (0° 90° 180° 270°) indicated with the red triangle at the center of the corresponding radial plots. After the adaptation period the adapting stimulus was removed and the rivalry targets were presented 45° preceding the adapting location. The rivalry target then cycled smoothly through the adaptation location for 2 min (12×10sec cycles). Together the plots show that adaptation to one of the rivalry targets will result in dominance of the non-adapted target, however, the spatial and temporal extent of this effect is limited and varies across subject and adaptation location.

Data from all four subjects showed that the local biases are nonexistent when the targets are moved very slowly around the periphery. This result is consistent with that reported by Blake et al (2003). However, contrary to their claim that no local biases in dominance exist, we show that such biases do exist if the target motion is fast enough (figure 3a–results for the 0.2Hz condition are not shown due to space limitations).

Experiment 4 was conducted to rule out the possibility that the lack of local bias during the slow cycle reflected a reduction in a “local memory effect” due to decay during the 100sec interval between each pass of the rivalry targets through a given location, rather than the extended duration of the rivalry target in each location. In order to distinguish between the effect of presentation duration and duration of elapsed time between successive presentations, the brief (1sec) intermittent paradigm of experiment 1 was used. Consistent with experiment 3, the target completed a cycle every 10, 20 and 100sec. Looking at the results from the 100sec condition, it is clear that location specificity is still seen at onset, even at local inter-stimulus intervals of up to 99sec. To further test the influence of perceptual memory and the stability of the onset bias, subjects were also re-tested 1–2 weeks later (Figure 3B). The fact that the local biases are still observed 1–2 weeks after the initial testing, suggests that perceptual memory is unlikely to play a major role in the observed biases. While memory may play some role, it appears that stable local factors are critical for the biases observed here.

The role of stable local factors in onset rivalry is clear, but the question remains whether anything can affect this onset bias. Blake et al., (2003) found that if the targets are made to move over an area that had previously been adapting to one of the rivaling target, it was possible to induce a switch favoring the non-adapted target. In experiment 5, the aim was to determine if it was similarly possible to induce a switch in perceptual dominance when the targets were moving fast enough to show the onset effect (10sec cycles). Two subjects from experiment 3 participated in this final condition. Each trial began with 60sec of adaptation to a stationary version of the non-dominant target (dominance was based on subjects reports in experiment 3–red gratings in both cases). In all other aspects the adaptor and target were identical. At the end of the adaptation period the adaptor was extinguished and the target was presented. The target always appeared in the location 45° preceding the place of adaptation and began moving immediately along the same circular trajectory used in experiment 3 (the targets passed over the adaptor location a total of twelve times in 2min). Subjects were asked to fixate for the entire duration of the trial and to report the perceptual dominance during the 2min binocular rivalry portion of the trial. Each subject participated in four trials with the adaptor being located in a different location for each trial (0°, 90°, 180°, 270°). As shown in figure 2c, in the first cycle over the adapted location the non-adapted target always became dominant but then generally switched immediately back after passing through the adapted region. In some cases this dominance pattern continued for the entire 2min, occasionally however, it was only the first one or two passes that were affected. An interesting observation is that the regions where the adaptation had the greatest effect over time (number of cycles) and space (spreading to neighboring regions) were the same regions that were most likely to be dominated by the non-adapted color in experiment 3. This implies that, while it might be possible to influence dominance, this influence acts relative to the underlying natural bias that already exists. The fact that the adaptation-induced dominance of the green target generally did not persist for the entire 12 loops, argues against the exclusive role of perceptual memory in the localized patterns of dominance observed in the previous trials.

## Discussion

This study aimed to demonstrate a single point–onset rivalry represents an early phase of perceptual competition that is distinct from sustained binocular rivalry. It is true that onset and sustained rivalry are both characterized by the exclusive dominance of one rivalry target at the expense of the alternative, temporarily suppressed, image. In this respect, the two states are phenomenologically identical. However, onset and sustained rivalry appear to be quite different in regards to the factors biasing dominance and may be driven, at least partially, by different underlying mechanisms.

In experiments 1 and 2, a brief (1sec) rivalry target was presented at 10sec intervals either in the fovea or in one of eight locations around the periphery. In all cases the dominance during the intermittent presentation was uncorrelated with the dominance during sustained rivalry. The most striking observation from these initial experiments was the location specificity and the magnitude of bias during the intermittent presentation. This location specificity could not be explained by regions of monocular dominance (eye-of-origin factors) or path history (sequential buildup of adaptation over a predictable trajectory across visual space). In experiment 3 the location specificity remained even when the targets were moved smoothly around the visual periphery. Interestingly, this location specificity broke down when the motion speed was reduced to speeds of 3.6°/sec, suggesting that the onset bias is sensitive to the presentation duration at any location and not “onset” per se. Experiment 4 used the intermittent (1sec) presentation to ensure that the lack of location specificity at slower speeds was, in fact, due to the reduction in speed and not the result of the increased intervals between subsequent target cycles. Using this paradigm, the strong local biases reappeared even when the inter-stimulus intervals were extended to 100sec (matching the slow smooth motion condition in experiment 3). Finally, we showed that periods of adaptation to one of the rivaling targets could induce local switches in dominance to the non-adapted target. However, these effects were generally limited to the spatial site of adaptation and had less influence over each subsequent cycle of the target (duration since removal of the adaptor). Together these results suggest that binocular rivalry at onset is a distinct phenomenon that is influenced by local factors independent of presentation eye and path history. Importantly, these local biases were found to be stable across periods ranging from 10sec to 2 weeks. The localized short term nature of the adaptation results suggests that, while the biases can be temporarily altered, the main factors appear to be stable local endogenous cues. “Perceptual memory” and other direct manipulations of the stimulus may (and likely do) impose some influence on the generation of onset dominance, however, these effects appear to play a lesser role.

The current results are particularly relevant to three recent studies that all reported perceptual stabilization with presentation times ranging from 0.5-1.2sec separated by offset durations of between 2-5sec [11], [13], [14]. These reports of perceptual stabilization were assumed to reflect the temporary suspension or slowing of the physiological processes underlying binocular rivalry. As a result, the finding that eye of presentation and location were the greatest factors in determining perceptual stabilization, was taken as evidence that perceptual memory is limited to these low-level factors [13], [14]. Using offset intervals of 10sec and beyond, we found no evidence of perceptual memory. While the lack of stabilization reported here appears to contradict these earlier studies, the results are not incompatible. Further experiments are needed to tease out the exact relationship between “onset” rivalry and perceptual memory. However, one possibility is that onset biases reflect stable endogenous differences in specific neural populations, while “recent perceptual history” may cause temporary changes in identical, overlapping or competing neural populations. On the basis of current results it is impossible to determine whether the influence of perceptual history is limited to the onset phase or if it represents a form of “physiological inertia” that is integral in driving perceptual transitions during sustained rivalry. In fact, one of the main conclusions of this paper is that brief presentation paradigms may be an inappropriate basis for any arguments about the mechanisms underlying sustained rivalry. If onset rivalry and sustained rivalry are influenced by different factors, the finding that local factors determine stabilization does not mean that higher level object-based factors do not play a critical role during normal sustained rivalry. Likewise, paradigms involving brief or intermittent stimulus presentation may not be suitable to confirm a role of higher-level factors such as “stored perceptual configuration” [11], [13], [14] or “object based attention” [15] in driving perceptual alternations during sustained viewing of ambiguous images.

Here we are claiming that onset rivalry is a distinct form of perceptual competition. Onset and sustained rivalry may involve identical mechanisms that differ only in their relative role in biasing dominance or their sensitivity to factors such as attention shown recently[16]. On the other hand the two forms of rivalry may share very little similarity, such that onset rivalry is more reminiscent of other paradigms such as “masking”[17]. While we are making no claims to the specific mechanisms involved, it is interesting to speculate about the existence of onset states in other forms of multi-stable stimuli. For example, the onset bias in binocular rivalry may be analogous to that reported with the plaid stimulus where the initial percept is almost exclusively dominated by a coherent image of fused gratings (a plaid) moving in a single direction. This is despite the fact that stimulus manipulations can be made to strongly bias the “ungrouped” percept of two sets of gratings sliding in opposite directions after the initial perceptual switch [18]. There appears to be factors about the “plaid” interpretation that facilitates it's dominance at onset that may not necessarily insure its dominance once rivalry has begun. It is hoped that future research aimed specifically at the earliest phases of rivalry may help tease out what the relevant factors are. Given that our visual input is always changing (as a result of saccadic eye movements and the dynamic nature of our visual environment), it is fair to suggest that the initial stages of perceptual competition are likely to be the most ecologically relevant. This is particularly true in the case of binocular rivalry. Sustained rivalry is an artificial creation of the lab, great for studying the processing of unselected material but not that relevant to everyday vision where brief rivalry due to monocular occlusions is seldom maintained by a viewer who can move his or her head for a unobstructed vantage point. Therefore, this initial rivalry phase, with very different properties from those of the sustained rivalry, is a far more appropriate measure of the outcome of actual rivalry occurrences in normal vision.

The fact that we do not get an inversion of results when the eyes are swapped suggests that the bias is not simply one of local regions of ocular dominance or retinal variations in color sensitivity. However, the location specificity observed suggests that the biases are likely to occur at a level at which retinotopic areas are clearly defined and stable over weeks, which is likely to be at a level “lower” than the frontal parietal networks that have been implicated in some rivalry models. It is worth noting that the location specificity observed in the onset biases did not show any systematic distribution across the visual hemi-fields. This result differs from the clear hemi-field asymmetries in switch rate and dominance durations found in normals [19] and spilt brain patients [20]. Given the minimal numbers used in the current study it is obviously possible that such hemi-field differences may exist when averaged over larger populations. However, on the basis of our results alone, it appears that the individual differences in the distribution of relative target dominance is one of the most striking features of onset dominance. If further testing continues to reveal large variation among observers in the spatial localization of the bias effect, these individual differences may end up offering an ideal access point through which to explore the different factors involved.

From the current set of experiments it is clear that binocular rivalry dominance is initially determined by low-level, spatially localized factors that are stable over periods of weeks. However, further experiments are needed to pinpoint, more exactly, the structures and mechanisms involved in onset rivalry. The implications of the proposed existence of onset rivalry raises questions about intermediate processes like flash suppression [21] or continual flash suppression [22] which occur when one image is presented continually while the other image is intermittently and repetitively flashed on for brief presentations. Furthermore, the illustrated phenomenological differences between onset and sustained rivalry should provide a cautionary footnote to future binocular rivalry investigations using brief presentation paradigms, particularly those making claims about biological mechanisms based on event related fMRI, EEG, MEG or single unit recordings. On a more positive note, we suggest that brief presentation paradigms are ideal for investigating inherent biases in visual and cognitive processing that are likely to be important for rapid percept formation during normal vision.

## Methods

### Experimental procedures

#### Participants

A total of 13 subjects participated in these experiments (7 Male and 6 female) aged between 20 and 33 participated in this study. An additional 4 subjects recruited for the study, were unable to participated because of their inability to experience definable perceptual dominance (the exclusion criteria for these individuals is outlined in detail below). All data collected from the 13 eventual participants is presented.

Apart from one of the authors (OC), all subjects were trained psychophysical observers but naïve to the aims of the experiment. All subjects reported normal or corrected to normal vision. These experiments were undertaken with the understanding and written consent of each subject, the review of the Federally mandated Harvard University Committee on the Use of Human Subjects in Research, and in conformation with The Code of Ethics of the World Medical Association (Declaration of Helsinki).

#### Stimulus and procedure

The stimuli were presented on a calibrated Apple Color monitor (1024×768 pixels, 75 Hz refresh) monitor viewed through a mirror stereoscope. Stimulus were programmed and presented using VisionShell^™TM^ software (MicroML, St. Hyacinthe, Canada). The stimulus background consisted of a uniform gray background (35cd/m^2^). During the foveal presentation used in experiment 1, the patches were either red (CIE x, y = .585, .332) and black or green (CIE x, y = .294, .574) and black gratings of 100% contrast (see figure 1a).

Responses were reported by key press on a standard computer keyboard with depression of the key signaling the dominance of the corresponding target. During intermittent conditions, subjects were asked to report the dominant percept after each 1sec presentation. In the case of sustained stimulus presentation, either in a single location for 60sec or during the smooth target motion, subjects were instructed to continually report the relative dominance of each of the two percepts. Subjects were instructed to focus on the target color and they were not given the opportunity to report mixed dominance states but were forced to indicate the dominance of one of the two targets (red or green–see below for more details).

#### Participant exclusion and stimulus manipulations

Eye of presentation was counter balanced for color and orientation across subjects but was kept constant within each subject. The experimental order and motion direction (clockwise and counter-clockwise) was also counterbalanced. Every trial was repeated once before the subsequent condition was tested. However, due to space limitation, here we present only responses for the initial trials.

Because one aim of the study was to consider the role perceptual memory or adaptation, we did not allow subjects to have a practice. Instead, responses were recorded from the initial stimulus presentation. After the completion of this initial trial (1–2 minutes, depending on the experiment), subjects were questioned in detail about their perceptual experience and their understanding of task. The majority of participants reported that the dominance during each presentation was very clear with either total or near-total dominance through the trial. The strength of dominance experienced was more in line with that achieved during flash suppression rather than the dynamic mixed states that are often experienced during rivalry transitions. A minority of 4 participants, however, reported experiencing a mixed state in which the relative dominance of the two targets ranged from 50:50 to approximately 30:70 on each presentation. These individuals described it as an indecipherable mixed state that was the dominant percept at nearly all locations for the entire trial. In an attempt to induce reportable dominance, people in this final group were presented with a variety of different stimulus conditions involving changes in relative stimulus strength and eye of presentation, multiple readjustments of the mirror stereoscope were also made. In all cases, these subjects were rejected from further part in the study after approximately 20minutes of unsuccessfully attempting to induce reportable dominance of either of the two stimuli. Because these 4 participants were unable to complete any of the experimental conditions they were not included in the participant description above.

Saturation of both targets was always kept at 100% for experiment 1. However, some minor changes to the target saturations were needed for some participants in the periphery intermittent presentation experiments (2&4). After the initial presentation of the experimental trial the phenomenological reports fell into two types 1) clear dominance during each one second presentation but alternating dominance between location–some in this final group spontaneously reported the location specificity of the relative dominance of the two rivaling colors (“the lower left patch is always green”), 2) exclusive dominance of one of two colors in every location on multiple cycles through the 8 locations. In this second case, the total dominance of one target for an entire trial was considered uninformative in respect to questions of both perceptual memory and location specificity. Therefore, to bring the stimulus closer to a point of equivalence, the saturation of the dominant target was successively reduced (e.g. the red of the red black patch was desaturated progressively toward gray) until the subject verbally reported experiencing dominance of the non-dominant target in at least one or two locations during each cycle of the stimulus (∼20% dominance for the non-predominant target). This saturation value was then used for all subsequent experiments that the individual participated in.

It should be noted that, despite the clear dominance, some participants reported a small but perceptual crescent of the non-dominant target around the target periphery. However, because the crescent was small and consistently present, participants were able to ignore it and focus on the central portion of the target while determining their responses.
